# Shareholder personal risk and firm risk: An empirical analysis of share pledges and firm debt policies

**DOI:** 10.3389/fpsyg.2022.1010162

**Published:** 2022-11-17

**Authors:** Jiahui Xia, Zhanchi Wu, Rongwu Zhang, Xiangyi Chen, Rui Zhang

**Affiliations:** ^1^School of Management, Jinan University, Guangzhou, China; ^2^School of Management, Guangzhou University, Guangzhou, China; ^3^Department of Economics, Jinan University, Guangzhou, China

**Keywords:** share pledge, debt policy, tunnel, personal risk, risk shifting, risk-taking, risky, ultimate owner

## Abstract

The impact of personal risk caused by controlling shareholders' equity pledges on the company's debt policy is an issue worth exploring. Using Chinese A-share listed companies from 2006 to 2020, this paper studies the impact of ultimate owner equity pledges on firm debt size and debt maturity structure and explores the mechanism of ultimate owner personal leverage on firms. The results show that the increase in ultimate owner stock pledges leads to higher financial leverage and a longer debt maturity structure for the company. In addition, the study reveals that the high personal leverage of the ultimate owner of the pledged equity is an influential mechanism driving the transfer of personal risk to the firm. In particular, even if a company's actual debt ratio is higher than its target debt ratio, equity pledges can prompt listed companies to increase their debt ratios and debt maturities, causing them to take on excessive debt risk and transfer the risk to creditors. It follows that the tunneling effect is a driving force of equity pledging and corporate debt policies. These results remain robust after the robustness test and endogenous test. The conclusions of this paper not only emphasize the impact of shareholders' personal risk on the firm but also provide a reference for investors' perception of firm risk.

## Introduction

Share pledging is a financial behavior where shareholders use their shares as collateral to borrow money from financial institutions. The share pledge of controlling shareholders is very common in emerging capital markets. In emerging markets such as India or China, insider pledging shares account for 35 to 50% of the listed companies (Dou et al., 2019). Specifically, controlling shareholder share pledges is especially popular in the mainland Chinese capital market (Xu and Huang, 2021). However, a higher proportion of share pledges may imply a higher personal financial risk. Whether the ultimate controller will transfer personal risk to the company in face of risk is a question worth exploring. In fact, in the Chinese market, the debt crisis of listed companies is often accompanied by a share pledge crisis. In particular, 2017 to 2020 saw a large number of outbreaks of controlling shareholder equity pledges by listed companies in mainland China, followed by debt defaults by the listed companies under their control^1^. Although prior studies have documented the influence of corporate insiders' behavior on corporate financial policy (Du et al., 2014; Dou et al., 2019; Puleo et al., 2021), this study explores how the share pledge of ultimate owner affects the debt risk of listed companies from the perspective of debt policy choices.

Existing studies have classified the impact of share pledges into two categories: tunneling effect and pressure effect. In detail, under the tunneling effect, a share pledge is an important way for shareholders to expropriate the company's assets. As a result, the share pledge aggravates the degree of separation of ownership and control of the company, which makes it convenient for shareholders to carry out tunneling. On the other hand, the pressure effect mainly comes from the “margin call” system of share pledges. If the stock price falls, the controlling shareholder may lose control of the company because of the failure to make up the margin. Therefore, to stabilize the stock price, the controlling shareholders are committed to improving the company's performance and market value management.

In this paper, we studied the mechanism of the impact of equity pledges on debt policy. The theoretical prediction of the direction of the effects is ambiguous. In detail, under the tunneling effect, ultimate owners tend to raise the leverage of the company to facilitate risk transfer and hope to control the right to “tunnel” the company after share pledges. However, ultimate owners also choose debt financing to prevent equity dilution due to the issuance of new shares and to further transfer the company's capital to reduce their liabilities or to repay the interest on the debt. Under the pressure effect, the ultimate owners may reduce the risk of stock price to avoid forced closing of the position and prefer a low-risk debt policy. With an equity pledge, the ultimate owners wish to reduce the company's leverage.

To study the relationship between share pledges and corporate debt policy, we used the annual observation samples from 2006 to 2020 in China's A-share market. We found that there was a significant association between the ultimate owner's share pledge and the company's debt policy and our empirical results supported the presence of the tunneling effect. Under the influence of “tunneling” motivation, the ultimate owner's share pledge rate is positively related to increases in the company's leverage. For debt maturity structure, we found that the ultimate owner after the share pledge tends to promote the company to choose long-term debt financing.

To further explore the impact of the ultimate owner's share pledge on the debt risk of listed companies, we examined whether the ultimate owner's influence on the debt policy of listed companies led to excessive risk-taking. We found that regardless of the company's financial situation having high leverage (the actual debt ratio exceeds the target debt ratio) or low leverage (the actual debt ratio is lower than the target debt ratio), the share pledge promoted the increase of the company's financial leverage. However, for the debt maturity structure, we found that the debt maturity of high-leverage companies increased with the share pledge rate of the ultimate owner, while that of the low-leverage companies had no significant change. This result confirms the risk-shifting motivation of controlling shareholders and that listed companies take excessive risk.

However, it is not difficult to think of a reverse causality explanation. For instance, firms with higher leverage and longer maturity might have better performance and higher valued shares, promoting ultimate owner to pledge shares. To alleviate concerns around the robustness of our results, we employed provincial average pledge rate as the instrumental variable to predict the leverage and maturity. The results of two-stage regression supported the baseline findings.

The main contributions of this study are as follows. First, this paper examines the impact of equity pledges on corporate risk-taking from the perspective of corporate debt policy. Our Findings further reveal that the tunneling effect is the main driving force in the internal mechanism of share pledge and debt policy. Second, this paper enriches the literature on the impact of shareholders' financial characteristics on their risky decisions. It also confirms the risk transfer hypothesis of the transmission of shareholders' individual risk to the firm, which provides external investors with further information on the true financial risk of the firm. Finally, our study enlarges the research on the economic consequences of equity pledges. Equity pledges are prevalent in emerging capital markets, and the Chinese market has its own financial characteristics and legal environment. Our study provides some reference value for regulators to formulate more effective policies.

The rest of the paper is organized as follows. Section Relevant literature and hypotheses development discusses the relevant literature review and proposes the main hypotheses. Section Data, variables, methods, and descriptive statistics describes the data, variables, as well as our research design. The main empirical findings are provided in Section Empirical analysis. Section Robustness tests conducts endogeneity and robustness tests. Section Conclusions and discussions offers the conclusion.

## Relevant literature and hypotheses development

### Relevant literature

#### Consequences of share pledge

Following Xu et al. (2019), we classified the impact mechanism of equity pledges into tunnel effect and pressure effect.

##### Tunneling effect

The tunneling effect is the phenomenon when the ultimate owners expropriate the companies' assets and transfer the benefits to themselves. For instance, Anderson and Puleo (2020) found that pledges of shares by influential insiders enable them to capture the private benefits of control at the expense of outside shareholders. In addition, previous studies have found that when the controlling shareholders pledge their shares, their company's innovation decreases (Ouyang et al., 2019; Pang and Wang, 2020), risk rises (Chen and Hu, 2007), performance becomes worse (Shin-Ping and Tsung-Hsien, 2009), and real earnings management occurs (Deren and Ke, 2018). Therefore, ultimate owners use share pledges to “tunnel” the company (Zhang et al., 2020), which is detrimental to the sustainability of the company.

##### Pressure effect

After a stock is pledged, if the company's share price falls triggering a margin call, the shareholder needs to replenish the margin in advance or redeem the pledged shares. If the shareholder fails to make up the margin in time or the share price falls below the closing line, the creditor will auction the pledged shares and the shareholder may face the risk of losing control of the company. The controlling shareholder fears losing control of the company and takes a series of measures to stabilize the share price, which is known as the pressure effect. Some scholars have demonstrated that equity pledges have a pressure effect. For example, during equity pledges, controlling shareholders' market capitalization management motivation is enhanced (Xie et al., 2016; Li et al., 2019), and they take the initiative to repurchase shares after equity pledges to counteract the potential risk of covering their positions (Chan et al., 2018). Moreover, companies with controlling shareholder equity pledges are more inclined to smooth their earnings (Huang and Xue, 2016) and have a more positive tone of disclosure (Zhao et al., 2019).

#### The conflicts between shareholders and creditors

There are significant differences in the preferences of creditors, shareholders, and management for debt policy. Many studies have examined debt policy from multiple perspectives, such as executives' risk preferences (Chava and Purnanandam, 2010; Cassell et al., 2012; Serfling, 2014), controlling shareholders' risk preferences (Hackbarth, 2009; Setia-Atmaja et al., 2009; Zeidan et al., 2018), and so on. In this paper, we examined the choice of debt policy from the perspective of the conflict between the ultimate owner and the creditor.

Shleifer and Vishny (1997) stated that the basic agency problem in most countries is the conflict between outside investors and controlling shareholders who have almost complete control over the managers. Meanwhile, controlling shareholders use corporate debt to deal with the Type II corporate agency conflict (De La Bruslerie and Latrous, 2012). In terms of financial leverage, Du and Dai (2005) found that the separation of cash flow rights and control leads to increased financial leverage in Hong Kong, Japan, and other emerging markets in East Asia (except the mainland Chinese market). The main driving force of this phenomenon is the non-dilution entrenchment effect, and Du and Dai (2005) expected that the tunneling effect would lead to a reduction in debt. In addition, Liu and Tian (2012) found that controlling shareholders in emerging markets with weak creditor protection raise funds through intercompany loans or related-party transactions after the reform of China's non-trading equity structure. For example, Tsai et al. (2015) found that middle shareholders and controlling shareholders collude to divert debt financing in Chinese state-controlled enterprises, and that state-owned shares play a decisive role in the collusion.

### Hypotheses development

A share pledge is a type of financing in which a shareholder uses stock as a pledge for a loan to himself or a company. The process of stock pledging is relatively simple for shareholders, and stock pledges have the advantages of low financing costs, fast lending, and flexible pledge terms and pledge rates. Moreover, a share pledge can bring more liquidity provided that the shareholder does not lose control of the business. When shares are pledged, the controlling shareholder's cash flow rights to the pledged shares are temporarily frozen, but the shareholder still has control over the company. This intensifies the degree of separation between the control and cash flow rights of the company, leading to an increase in the control leverage of listed companies and providing the possibility for shareholders to pursue excessive profits. Therefore, as rational economic agents pursuing profits, controlling shareholders use equity pledge financing to relieve themselves when they encounter financial difficulties (Zheng et al., 2014).

In many countries, the ownership structure of companies is highly concentrated, and controlling shareholders tend to grab the private benefits of control (Grossman and Hart, 1980), which is the portion of corporate value enjoyed by the party in control (Dyck and Zingales, 2004). Thus, the ultimate controlling shareholder has significant influence over the affairs of the company by creating a complex chain to control the management of the listed company.

Notably, the quality of legislative protection for creditors affects the choice of debt policy by firms. For example, section 135 of the Companies Act 1985 in America ensures that the interests of creditors are protected through a return of surplus capital (Armour, 2000). This surplus system can be seen as an important measure to protect creditors. However, the Chinese market usually uses corporate credit to measure the solvency of a company and does not consider the liquidity of the company. Dynamic corporate solvency standards have not yet been established (Feng, 2019), and as a result, it is difficult for creditors to make accurate judgments about the actual solvency of the company. Therefore, the ultimate owner can avoid the supervision of strong creditors when making decisions about the company's finances (Liu et al., 2015). However, based on the different mechanisms of influence of equity pledges, the ultimate controlling shareholders may have different preferences for debt selection. The specific analysis is as follows:

#### Tunneling effect on leverage

The increased leverage of control following a share pledge facilitates the controlling shareholder's choice of corporate debt policy, and the lack of legislative protection provides a natural environment for controlling shareholders to infringe on the interests of creditors. Thus, in the choice of debt policy, controlling shareholders have the ability and incentive to tap the private benefits of control (Grossman and Hart, 1988). For example, shareholders hold more control with less ownership and have an incentive to push the firm to borrow money to invest in risky projects (Jensen and Meckling, 1976). In turn, when the firm's investment projects generate large amounts of income, shareholders receive most of the income over the book value of the liabilities. However, when the firm's investment projects fail, shareholders are limited only by the liabilities, while creditors are not guaranteed interest income and may even suffer a loss of principal. Moreover, although companies can raise equity financing, stock issues can dilute shareholders' equity and affect their control. Corporate debt, however, does not directly affect shareholder control. At the same time, debt financing gives shareholders control over assets far over the shares they actually invest. Thus, the firm provides a risk barrier for shareholders, acting as a buffer against direct creditor liability to shareholders. Chen and Hu (2007) supported the risk transfer effect by arguing that under conditions of high leverage, the personal wealth of shareholders may be less than their liabilities, and therefore the adverse effects of risk are less likely to affect large shareholders. In addition, Chen and Hu (2007) demonstrated that the level of personal debt of controlling shareholders is positively related to the level of risk in listed companies, but negatively related to the performance of the company.

Equity pledges often occur when shareholders are experiencing financial difficulties. To alleviate financial pressures, shareholders usually use related transactions and other benefit transfer practices to expropriate company property. To ensure the smooth implementation of tunneling, shareholders have the incentive to push the company's leverage. The ultimate owner controls the debt financing of the listed company to gain more controllable resources (Sun, 2008). Based on the tunneling effect, shareholders are likely to use listed companies as a debt financing platform to transfer their financial risk to listed companies and then to creditors. Therefore, we propose the following hypothesis:

**H1a:** The increase of equity pledges by ultimate owners promotes listed companies to increase their financial leverage.

#### Pressure effect on leverage

However, there may be competing views in the theoretical analysis. Due to the “margin call” system, if the controlling shareholder cannot make up the margin in time, the pledged shares will be forced to close out and the controlling shareholder will face the risk of losing control of the company. When the share price falls below the closing line, although the shareholder has the motive to “tunnel,” it will have a significant impact on the shareholder's assets if the stockholder loses control of the company because of the stock pledge.

After shares are pledged, if the share price falls or even hits the warning line, the shareholder may need to sell or pledge other unpledged shares or physical assets to increase security or redeem the shares early. Further, when the share price falls to the closing line, the pledgee will auction the shares at a market price lower than the share price at the time of the pledge to exercise the rights of creditors and reduce losses. If the auction price is not sufficient to repay the shareholder's debt, then the shareholder who pledged the stock still has to cover the debt. This result sends a negative signal to the market about a shortage of capital, and external investors may lower the stock price further. When outside investors are negative about the company and the stock, there is more pressure for the stock price to fall and the market value of the company to decrease, thus putting more pressure on controlling shareholders who are already in deep financial trouble. What's more, if shareholders do not cover their debts or provide additional guarantees, the pledged shares are at risk of being forced to close out, threatening the shareholders' control. If the company introduces strategic investors or issues new shares to avoid the risk, the controlling shareholder's equity is diluted, exacerbating the risk of control transfer.

Under pressure from the risk of transfer of control, controlling shareholders may urge companies to adopt safer, less leveraged debt policies. A company that raises debt on a large scale creates a high debt overhang, then the high leverage forces the company to be obligated to repay various debts. Dou et al. (2019) found that margin calls triggered by price crashes exacerbate the risk of bankruptcy of pledged firms. Thus, if a firm's solvency declines and it is unable to repay its loans, it may face debt restructuring or even bankruptcy liquidation. In addition, the business condition and value of the company are important factors affecting the stock price. External investors believe that both the controlling shareholder and the company are in a highly leveraged and risky position after the equity pledge. Risk-averse rational investors tend to sell the company's stock, putting pressure on the company's stock price, thereby adversely affecting the ultimate owner's control. Therefore, we formulate the competitive hypothesis:

**H1b:** The increase in share pledges by ultimate owners contributes to a reduction in the company's financial leverage.

## Data, variables, methods, and descriptive statistics

### Sample selection

We considered all China A-share listed companies from 2006 to 2020 and classified the industries according to the Guidelines on the industry classification of listed companies (revised in 2012) published by the China Securities Regulatory Commission (CSRC). In addition, data on stock pledges were obtained from the WIND database. Moreover, the governance structure data of listed companies were taken from CCER Data. Besides, the basic information, financial data, and stock trading data of listed companies were obtained from the CSMAR database.

We removed financial and insurance companies from this sample and we also removed data relating to ST (special treatment) companies. Further, we removed companies with missing data items on key variables. For the sample with multiple pledges from the same ultimate owner in the same year, the sample size was calculated only once to avoid duplicate values. Consistent with previous empirical studies, we removed samples that were released in the same year after being pledged. After filtering, the observed values of non-missing values for the key variables *LEV* (financial leverage), *PledgeR* (share pledge ratio of the ultimate owner), and control variables were 18,899. We used Stata16 to process the data in this paper.

### Variables

#### Share pledge ratio of the ultimate owner

We used the share pledge ratio of the ultimate owner as our key explanatory variable (*PledgeR*). This variable was constructed as the ratio of the sum of shares pledged by the ultimate owner to the sum of shares owned by the ultimate owner at the end of year t.

#### Financial leverage

We used the book leverage ratio as our key explanatory variable. It was constructed as the ratio of the book value of total liabilities to the book value of total assets. Further, we used an incremental variable to measure the financial leverage ratio. That is, we calculated the difference in financial leverage between year t+1 and year t to obtain the financial leverage variable, denoted by *LEV*.

#### Control variables

Consistent with previous studies (Barclay and Smith, 1995; Guedes and Opler, 1996), we used a set of control variables: firm size (*Size*), growth capacity (*Growth*), firm value (*TobinQ*), firm profitability (*ROE*), the ratio of expenses to sales (*SI*), the shareholding ratio of the largest shareholder (*First*), firm's non-debt tax shield (*NDA*), firm's currency stock (*Cash*), and firm's liquidity (*LRate*). In addition, we set the year dummy variable (*Year*) and industry dummy variable (*Industry*) to measure the year effect and industry effect, respectively. Among them, we set industry dummy variables according to the industry classification of the CSRC 2012 edition.

Details of variable definitions are listed in Table 1.

**Table 1:** Variable definitions.

**Variable**	**Definition**
ΔLEV	LEV = total liabilities/total assets, ΔLEV_t+1_ = LEV_t+1_-LEV_t_
PledgeR	The sum of shares pledged by the ultimate owner divided by the sum of shares owned by the ultimate owner at the end of year t.
Size	The natural logarithm of the total market value.
Growth	The change in operating income between year t and year t+1, measured by the operating income at the beginning of year t.
TobinQ	The ratio of the sum of total market value and total liabilities of the company in year t to total assets in the previous period.
ROE	Net income divided by equity.
SI	Selling expenses divided by operating income.
First	The number of shares held by the largest shareholder scaled by the total number of shares.
NDA	Accumulated depreciation divided by total assets.
Industry	An indicator variable that is a dummy variable to measure industry effect.
Year	An indicator variable that is a dummy variable to measure year effect.

### Empirical specifications

In this paper, we measured the financial leverage in year t+1 and regressed it on share pledge variable measured in year t along with several control variables in year t. We constructed the following model for regression:


(1) $$\begin{array}{l} \Delta LEV_{it+1}=\alpha +\beta_{1}PledgeR_{it}+\beta_{2}Size_{it}+\beta_{3}Growth_{it}\\ \;+\beta_{4}ROE_{it}+\beta_{5}TobinQ_{it}+\beta_{6}SI_{it}+\beta_{7}First_{it}\\ \;+\beta_{8}NDA_{it}+\beta_{9}Industry+\beta_{10}Year+\varepsilon_{it} \end{array}$$

In Model 1, Δ*LEV*_*it*+1_ was constructed by the difference of book-leverage value between year t and year t+1. *PledgeR*_*it*_ was the explanatory variable and represents the ratio of share pledge by ultimate owner. *Size*_*it*_, *Growth*_*it*_, *ROE*_*it*_, *TobinQ*_*it*_, *SI*_*i*_, *First*_*it*_, and *NDA*_*it*_ were control variables explained in Table 1. We controlled for the year and industry-fixed effects.

Specifically, we examined the effect of the ultimate owner's equity pledge on the size of the firm's debt. Further, we tested the effect of ultimate controller equity pledges on the maturity structure of firm debt. In addition, to explore the mechanism of action between shareholder equity pledges on a firm's debt policy, we examined the association between the high leverage of the ultimate owner and the firm's financial leverage and debt maturity structure. In addition, to avoid endogeneity issues, we used instrumental variables for testing. Finally, we used alternative variables for robustness testing.

### Descriptive statistics

Table 2 provides descriptive statistics for the variables used in the study. The average book leverage, *LEV*, was 49.85%, but the maximum was 84.19% and the minimum was 3.72%. The large range indicates that there was a wide variation in the debt ratios of listed companies. The mean value of the ultimate owners' stock pledge ratio, *PledgeR*, was 31.65%. In addition, *PledgeR* had a maximum value of 1 and a minimum value of 0, which implies that the ultimate owners are under different capital pressures.

**Table 2:** Descriptive statistics.

**Variable**	***N***	**Mean**	**Median**	**Max**.	**Min**.	**Std. dev**.
LEV	18,899	0.4985	0.4685	0.8419	0.0372	0.2030
PledgeR	18,899	0.3165	0.0000	1.0000	0.0000	0.5110
Size	18,899	22.1414	22.0785	28.5590	18.4019	1.1131
Growth	18,899	0.4771	0.4510	1.6328	−0.9972	0.2459
ROE	18,899	0.0682	0.0786	0.3190	−0.4295	0.1051
TobinQ	18,899	2.7201	1.9460	10.0135	0.9023	4.5410
SI	18,899	0.0708	0.0360	2.2730	0.0000	0.0727
First	18,899	0.3584	0.3357	0.8999	0.0029	0.1554
NDA	18,899	0.0240	0.0197	0.6767	−0.0030	0.0218

Provides descriptive statistics for various characteristics of the sample firms. Variable definitions are provided in Table 1.

## Empirical analysis

### Multiple regression analysis

To assess the effect of the ultimate owner share pledge ratio on the financial leverage of listed companies, we selected the incremental book-leverage (Δ*LEV*) of stage t+1 as the explained variable and empirically tested H1. Table 3 provides the regression results.

**Table 3:** The association between share pledge of ultimate owner and firm's financial leverage.

	**(1)**	**(2)**
**Variables**	**ΔLEV_t+1_**	**ΔLEV_t+1_**
PledgeR_t_	0.0527^***^	0.1328^***^
	(4.115)	(3.723)
Size_t_		−0.0610
		(−1.060)
Growth_t_		−0.0006
		(−0.720)
ROE_t_		−0.0018
		(−0.086)
TobinQ_t_		−0.0641^***^
		(−3.682)
SI_t_		−0.0354
		(−0.711)
First_t_		−0.5334
		(−1.119)
NDA_t_		2.0374
		(0.614)
Constant	0.0633^***^	1.6555
	(2.944)	(1.233)
Industry	Yes	Yes
Year	Yes	Yes
*N*	18,899	18,899
*R* ^2^	0.105	0.367

Robust t-statistics adjusted for firm level clustering are given in brackets.

*** denotes the coefficient is significant at the 1% level.

First, we did not consider the control variables and column (1) shows the results of the regression. We found that the *PledgeR*_*t*_ coefficient is 0.0527, which is significantly positive at the 1% level. In column (2), the coefficient of *PledgeR*_*t*_ increases to 0.1328, which is significantly positive at the 1% level. This indicates that the share pledge ratio of the ultimate owner is significantly and positively related to the leverage ratio. As stock pledges increase, listed companies can tend to adopt a higher financial leverage policy. Therefore, this result is consistent with H1a, while rejecting H1b.

### Further analysis

#### Share pledge and debt maturity structure

Under the tunnel effect, equity pledges allow the ultimate owner to have excess control. Since cash liquidity is very important to shareholders, short-term debt exposes the company to relatively frequent repayment status, which is not conducive to the stability of the high control leverage status of the ultimate owner. In contrast, relatively long debt maturities favor the implementation of high-risk investments, related transactions, and other actions by the ultimate owner.

In Diamond's (1991) model, short-term debt exposes the firm to the risk of excessive creditor liquidation. If there is a temporary adverse cash flow shock, the firm may not be able to borrow against future cash flows. Such a firm with excessive short-term debt faces a higher probability of bankruptcy than a firm with long-term loans. In less extreme cases, short-term debt exposes a company to considerable refinancing and interest rate risk, which can lead to higher earnings volatility. With both effects, the ultimate owner and firms prefer a safer long-term debt financing policy in terms of risk aversion. As a result, we supposed higher equity pledges by ultimate owners to drive longer debt maturities for listed companies.

The debt maturity structure is one of the explained variables in this paper. Following Barclay and Smith (1995), we constructed this measure as the ratio of long-term debt to the sum of short-term and long-term debt. Similarly, we used an incremental variable to measure the debt maturity structure. That is, we calculated the difference in the value of the debt maturity structure between years t+1 and t to obtain the debt maturity structure variable, denoted by Δ*DebtS*.

Similarly, we measured the debt maturity structure in year t+1 and regressed it on the equity pledge variable measured in year t, as well as on several control variables in year t. The model was as follows:


(2) $$\begin{array}{l} \Delta DebtS_{it+1}=\alpha +\beta_{1}PledgeR_{it}+\beta_{2}Size_{it}+\beta_{3}ROE_{it}\\ \;+\beta_{4}Growth_{it}+\beta_{5}Cash_{it}+\beta_{6}NDA_{it}+\beta_{7}First_{it}\\ \;+\beta_{8}LRate_{it}+\beta_{9}Industry+\beta_{10}Year+\varepsilon_{it} \end{array}$$

In Model 2, Δ*DebtS*_*it*+1_ represents the financial leverage of firm i in year t+1, which consists of the difference between the values of debt maturity structure in years t+1 and t. *PledgeR*_*it*_ is the explanatory variable and represents the ratio of share pledge by ultimate owner. *Size*_*it*_, *ROE*_*it*_, *Growth*_*it*_, *NDA*_*it*_, and *First*_*it*_ are the control variables explained in Table 1. *Cash*_*it*_ is calculated by the amount of currency stock scaled by total assets. *LRate*_*it*_ is calculated by current assets divided by current liability. We included year fixed effects and industry fixed effects. Table 4 provides the results of the regressions.

**Table 4:** The association between share pledge of ultimate owner and firm's debt maturity structure.

	**(1)**	**(2)**
**Variables**	**ΔDebtS_t+1_**	**ΔDebtS_t+1_**
PledgeR_t_	0.0462**	0.0461**
	(2.591)	(2.717)
Size_t_		−0.0012
		(−0.945)
ROE_t_		0.0039
		(1.159)
Growth_t_		0.0001
		(1.520)
Cash_t_		−0.0014
		(−1.672)
NDA_t_		−0.4013***
		(−5.566)
First_t_		−0.0105
		(−1.489)
LRate_t_		−0.0094***
		(−4.842)
Constant	−0.0028	0.0775***
	(−0.367)	(3.456)
Industry	Yes	Yes
Year	Yes	Yes
*N*	14,797	14,797
*R* ^2^	0.155	0.331

Robust t-statistics adjusted for firm level clustering are given in brackets. *** and ** denote the coefficient is significant at the 1 and 5% level, respectively.

Column (1) of Table 4 shows the results of the regression without considering the control variables. The coefficient of *PledgeR*_*t*_ is 0.0462, which is significantly positive at the 5% level. In column (2), the coefficient of *PledgeR*_*t*_ is 0.0461, which is significantly positive at the 5% level. These findings showed that the share pledge ratio of the ultimate owner has a significant positive impact on the debt maturity structure. Thus, the results based on *PledgeR*_*t*_ suggest that as the share pledge ratio increases, listed companies can tend to have longer debt maturities.

#### Does the high leverage of major shareholders lead to excessive risk-taking by listed companies?

Equity pledges by major shareholders exacerbate the debt financing risk of listed firms. However, does this lead to excessive debt risk-taking by listed firms? Dong et al. (2010) found that excessive risk-taking leads to deviation from the target capital structure and reduces the maximum value of the firm. This is both detrimental to the interests of creditors and inconsistent with the long-term interests of minority shareholders. In this section, we further investigated the differential impact of pledging major shareholders' shares on the debt financing policies of firms with different debt profiles.

While the interests of major shareholders are closely tied to their shares in listed companies, the impact of personal debt risk is usually limited to their own wealth. However, in the presence of high leverage and plummeting stock prices, shareholders' personal wealth may be lower than their debts. Under the risk transfer effect, the adverse aspects of the risk weaken the impact on the financial position of the majority shareholder and increase the impact on the financial position of the firm. Thus, high shareholder leverage may lead to excessive risk-taking by public companies. Nevertheless, prior research has shown that the risk of control transfer is exacerbated by increased share pledges due to the impact of margin call systems (Zhu et al., 2021). Faced with the pressure of losing control, the ultimate owner may transfer personal risk to the firm. In addition, when a listed company's own debt risk is high, the ultimate owner's transmission of debt risk to the listed company may result in a default on the listed company's debt and thus loss of control, which may discourage shareholders' incentive to transmit debt risk to the listed company.

Next, we analyzed how the high leverage of listed companies affects the risk-shifting behavior of major shareholders—through the risk-shifting effect or avoiding the risk of control transfer. Specifically, we classified listed companies into high and low debt groups based on the deviation of the actual debt ratio from the target debt ratio. We investigated the effect of the high leverage of large shareholders on financial leverage policy and debt maturity policy. If the risk-shifting effect dominates, the high leverage of large shareholders tends to promote listed companies to improve their debt ratios and maturity structures, regardless of whether the actual debt ratios of listed companies are higher than the target debt ratios. If the effect of control transfer risk dominates, the pledge ratio of large shareholders only affects the debt policy of low-debt firms.

Previous studies have mainly used earnings volatility to measure the level of risk-taking of listed firms (Boubakri et al., 2013; Kusnadi, 2015). Following Hovakimian et al. (2001), we observed that listed companies continue to increase their leverage in the presence of high debt, i.e., the target debt ratio of listed companies is smaller than the actual debt ratio, as a way to determine whether the company has excessive risk-taking. When a listed company is highly indebted, the equity pledge ratio of major shareholders is positively correlated with the financial leverage and debt maturity of the company, indicating excessive risk-taking behavior of the listed company.

We constructed the following regression model:


(3) $$\begin{array}{l} Lev_{it}=\alpha_{1}ROE_{it}+\alpha_{2}BM_{it}+\alpha_{3}R_{it}+\alpha_{4}SI_{it}+\alpha_{5}TAR_{it}\\ \;+\alpha_{6}Size_{it}+\eta_{it} \end{array}$$

In Model 3, *Lev*_*it*_ represents the asset-liability ratio of firm i in year t, which is calculated by book liabilities divided by the sum of book liabilities and market value of stocks. *BM*_*it*_ is the book-to-market value of firm i in year t. *R*_*it*_ is the average stock return of firm i in years t+1 and t. *TAR*_*it*_ denotes the tangible asset ratio of firm i in year t and is constructed as the ratio of tangible assets to total assets.

We used Model 3 to calculate the firm's target debt ratio, which is the fitted value of *LEV*. We signed it with *Lev*<*uscore*>*hat*. When the target debt ratio is less than the actual debt ratio, the listed company is a high-debt company; when the target debt ratio is higher than the actual debt ratio, the listed company is a low-debt company. We employed *LD* dummy variable to measure the deviation of the listed company from the target debt ratio. *LD* equals to 1 if the listed company is a high-debt company, otherwise, it equals 0.

As previously discussed, this paper used Model 1 and Model 2 to test the impact of large shareholders' equity pledges on the financial leverage and debt maturity structure of listed companies, respectively. To further analyze whether equity pledges by the ultimate owners under their high leverage led to excessive risk-taking by listed companies, we ran separate regressions for the group of high and low-debt firms. We estimated the significance of coefficient β_1_ in Model 1 and Model 2 when *LD* equals 1 or 0. We expected that the risk transfer effect plays a dominant role in excessive risk-taking of the firm when β_1_ is significantly positive for the high debt group or both high and low debt groups and that the risk of control transfer dominates excessive risk-taking of the firm when β_1_ is significantly positive for the low debt group.

We selected the incremental debt financing scale in period t+1, Δ*Lev*_*t*+1_, as the explanatory variable in Model 1. In addition, we controlled for industry and year-fixed effects. Table 5 shows the results of the regressions.

**Table 5:** The association between high leverage of ultimate owner and firm's financial leverage.

	**High debt firm**		**Low debt firm**	
	**(1)**	**(2)**	**(3)**	**(4)**
**Variables**	**ΔLEV_t+1_**	**ΔLEV_t+1_**	**ΔLEV_t+1_**	**ΔLEV_t+1_**
PledgeR_t_	0.0467**	0.1491***	0.1026**	0.0911*
	(2.349)	(3.834)	(2.283)	(2.045)
Size_t_		−0.0725		−0.0005
		(−1.002)		(−0.385)
Growth_t_		−0.0008		−0.0007
		(−0.701)		(−1.272)
ROE_t_		0.0018		0.0227
		(0.094)		(1.164)
TobinQ_t_		−0.0757***		0.0063***
		(−6.168)		(3.221)
SI_t_		−0.0401		−0.0118
		(−0.702)		(−1.156)
First_t_		−0.6991		0.0004
		(−1.150)		(0.030)
NDA_t_		2.7085		−0.2602***
		(0.682)		(−3.423)
Constant	0.0650**	1.9791	0.0547***	0.0627**
	(2.399)	(1.166)	(6.213)	(2.151)
Industry	Yes	Yes	Yes	Yes
Year	Yes	Yes	Yes	Yes
*N*	14,299	14,299	2,792	2,792
*R* ^2^	0.172	0.241	0.115	0.375

Robust t-statistics adjusted for firm level clustering are given in brackets. ***, **, * denote the coefficient is significant at the 1, 5, and 10% level, respectively.

In Table 5, columns (1) and (2) present the regression results for the group of highly indebted firms. Column (1) is the regression result without considering the control variables. The coefficient of *PledgeR*_*t*_, the share pledge ratio of the ultimate owner, is 0.0467, which is significantly positive at the 1% level. In column (2), the coefficient of *PledgeR*_*t*_ rises to 0.1491, which is significantly positive at the 1% level. Columns (3) and (4) of Table 5 present the regression results for the group of low-debt firms. Column (3) is the regression result without considering the control variables. The coefficient of *PledgeR*_*t*_ is 0.1026, which is significantly positive at the 5% level. In column (4), the coefficient of *PledgeR*_*t*_ is 0.0911, which is significantly positive at the 10% level.

From the above results, we found that the share pledge ratio of the ultimate owner has a significant positive effect on the financial leverage of the company regardless of whether the listed company is at a high leverage level or not. As the share pledge ratio of the ultimate owner increases, the financial leverage of the listed company also increases. Hence, the above results verified that the risk transfer effect dominates the excessive risk-taking of the firm.

We next analyzed the impact of the ultimate owner-share pledge ratio on the debt maturity structure of listed companies at different leverage levels. As in the above study, we classified listed companies into high and low debt groups based on whether their actual gearing ratios are higher than the target debt ratios. We chose the incremental debt maturity structure in period t+1, Δ*DebtS*_*t*+1_, as the explanatory variable. We also controlled for industry and year-fixed effects. Table 6 provides the regression results.

**Table 6:** The association between high leverage of ultimate owner and firm's debt maturity structure.

	**High debt firm**		**Low debt firm**	
	**(1)**	**(2)**	**(3)**	**(4)**
**Variables**	**ΔDebts_t+1_**	**ΔDebts_t+1_**	**ΔDebts_t+1_**	**ΔDebtS_t+1_**
PledgeR_t_	0.0692***	0.0702***	0.0098	0.0319
	(3.158)	(3.242)	(0.145)	(0.637)
Size_t_		−0.0021		0.0030
		(−0.997)		(0.421)
ROE_t_		0.0038		0.1440**
		(1.160)		(2.471)
Growth_t_		0.0002		0.0008
		(1.233)		(1.524)
Cash_t_		−0.0012		−0.0069*
		(−0.808)		(−1.843)
NDA_t_		−0.3204***		−0.9746***
		(−3.721)		(−4.295)
First_t_		−0.0093		0.0094
		(−1.156)		(0.462)
LRate_t_		−0.0105***		−0.0098*
		(−7.951)		(−2.004)
Constant	0.0005	0.0923***	−0.0085	0.0983
	(0.069)	(3.191)	(−0.730)	(0.792)
Industry	Yes	Yes	Yes	Yes
Year	Yes	Yes	Yes	Yes
*N*	11,850	11,850	1,896	1,896
*R* ^2^	0.113	0.275	0.193	0.323

Robust t-statistics adjusted for firm level clustering are given in brackets. ***, **, * denote the coefficient is significant at the 1, 5, and 10% level, respectively.

In Table 6, columns (1) and (2) show the regression results for the group of highly indebted firms. Column (1) is the regression result without considering the control variables. The coefficient of *PledgeR*_*t*_, the share pledge ratio of the ultimate owner, is 0.0692, which is significantly positive at the 1% level. In column (2), the coefficient of *PledgeR*_*t*_ rises to 0.0702, which is significantly positive at the 1% level. Columns (3) and (4) present the regression results for the group of companies with low debt. Column (3) is the regression result without considering the control variables. The coefficient *PledgeR*_*t*_, is 0.0098, but it is not significant. In column (4), the coefficient of *PledgeR*_*t*_ is 0.0319, which is also insignificant.

From the above results, we found that when listed companies are at high leverage levels, the share pledge ratio of the ultimate owners has a significant positive effect on the debt maturity structure of the company. With the increase of the share pledge ratio of the ultimate owner, the debt maturity of listed companies tends to be longer. However, when listed companies are at low leverage levels, the ultimate owner's share pledge ratio has no significant effect on the debt maturity structure of listed companies. Thus, these results further supported the hypothesis that risk-shifting effects play a dominant role in firms' excessive risk-taking.

## Robustness tests

### Endogeneity: Instrumental variables methods

To alleviate concerns about the robustness of our results, we used a two-stage least square (2SLS) approach to address the endogeneity problem caused by reverse causality. Following Xie et al. (2016), we selected the provincial average pledge rate in year t, *ProPledgeR*, as an instrumental variable for the pledge of the ultimate owner's shares.

We first examined the effect of the average provincial pledge ratio on the share pledge ratio of the ultimate owners. Then, we investigated the effect of the average provincial pledge rate on the financial leverage and debt maturity structure of listed firms. Finally, we added instrumental variables to Models 1 and 2 to test the impact of ultimate owners' share pledges on listed firms' debt policy choices. Following Giroud et al. (2012), in the first stage of regression, we used Model 4 to investigate the effect of the average provincial pledge rate on the share pledge rate of the ultimate owners of listed companies.


(4) $$\begin{array}{l} PledgeR_{i}=\alpha +\beta ProPledgeR_{i}+\gamma X_{i}+\varepsilon_{i} \end{array}$$

In Model 4, i represent i listed company in the A-share market. α is a constant Term. *PledgeR*_*i*_ is the share pledge ratio of the ultimate owner of company i. *ProPledgeR*_*i*_ represents the average pledge rate of the province where company i is located. *X* stands for a set of firm-specific control variables proven to be associated with *PledgeR*_*i*_. ε is the residual error.

Next, we applied the results of the first stage to the second stage. We expressed the explanatory variable *PledgeR*_*i*_ by Model 4 and substituted it into Model 1 and Model 2 to investigate the effect of ultimate owner share pledge ratio on the financial leverage and debt maturity structure of listed companies. Table 7 shows the regression results of 2SLS.

**Table 7:** The regression results of 2SLS.

	**Financial leverage**		**Debt maturity structure**	
	**First stage**	**Second stage**	**First stage**	**Second stage**
**Variables**	**PledgeR_t_**	**ΔLEV_t+1_**	**PledgeR_t_**	**ΔDebtS_t+1_**
PledgeR_t_		0.1597***		0.2621**
		(3.05)		(2.14)
ProPledgeR_t_	0.1251***		0.1179***	
	(10.62)		(8.51)	
Size_t_	0.0012		0.0001	
	(0.73)		(0.06)	
ROE_t_	0.0018		0.0019	
	(1.19)		(1.14)	
Growth_t_	0.0012**		0.0013**	
	(2.16)		(2.15)	
Cash_t_	−0.0025**		−0.0031**	
	(−2.35)		(−2.46)	
Age_t_	−0.0125***		−0.0180***	
	(−8.35)		(−7.79)	
First_t_	0.0035		0.0025	
	(0.48)		(0.27)	
SI_t_		0.0212**		
		(2.10)		
TobinQ_t_		−0.0014		
		(−1.15)		
NDA_t_		−0.3047***		−0.4563***
		(−4.49)		(−4.12)
LRate_t_				−0.0086***
				(−2.97)
Constant	0.0511**	0.0042	0.1030***	0.0207**
	(2.02)	(0.99)	(3.54)	(2.25)
Industry	Yes	Yes	Yes	Yes
Year	Yes	Yes	Yes	Yes
*N*	5,575	5,575	3,488	3,488
*R* ^2^	0.475	0.076	0.448	0.072

Robust t-statistics adjusted for firm level clustering are given in brackets. *** and ** denote the coefficient is significant at the 1 and 5% level, respectively.

For the regression results of financial leverage, in the first stage, the coefficient of regression on the provincial average pledge ratio, *ProPledgeR*_*t*_, is 0.1251, which is significantly positive at the 1% level. In the second stage, the coefficient of *PledgeR*_*t*_ is 0.1597, which is significantly positive at the 1% level. In addition, for the regression results of debt maturity structure, in the first stage, the coefficient of *ProPledgeR*_*t*_ is 0.1179, which is significantly positive at the 1% level. In the second stage, the coefficient of *PledgeR*_*t*_ is 0.2621, which is significantly positive at the 1% level. These regression results are consistent with the previous results. From the above results, we found that even after we address the endogeneity of share pledges using the instrumental variables approach, share pledges by ultimate owners still have a significant positive effect of firms' financial leverage and debt maturity structure.

### Alternative measure

#### Alternative measure of financial leverage

To figure out whether the equity pledge of the ultimate owner affects the debt financing of listed companies, we replaced the measure of financial leverage to test its robustness. Following Hovakimian et al. (2001), we used the ratio of debt to market value as a proxy variable for financial leverage. We adopted an incremental variable to express the financial leverage variable. This variable consists of the difference between the financial leverage in year t+1 and the financial leverage in year t, denoted by Δ*Lev*. Table 8 shows the regression results for Model 1.

**Table 8:** Robustness: the regression results of changing financial leverage variables.

	**(1)**	**(2)**
**Variables**	**ΔLEV_t+1_**	**ΔLEV_t+1_**
PledgeR_t_	0.0207***	0.0282***
	(4.329)	(6.561)
Size_t_		0.0153***
		(15.126)
Growth_t_		0.0001
		(0.872)
ROE_t_		0.0022
		(1.662)
TobinQ_t_		0.0004*
		(1.802)
SI_t_		−0.0033
		(−1.384)
First_t_		−0.0046*
		(−2.008)
NDA_t_		−0.1636***
		(−8.075)
Constant	0.0771***	−0.2427***
	(24.408)	(−11.980)
Industry	Yes	Yes
Year	Yes	Yes
*N*	18,318	18,318
*R* ^2^	0.475	0.491

Robust t-statistics adjusted for firm level clustering are given in brackets. *** and * denote the coefficient is significant at the 1 and 10% level, respectively.

In Table 8, column (1) shows the results of the regression without considering the control variables. In column (1), the coefficient of *PledgeR*_*t*_, is 0.0207, which is significantly positive at the 1% level. In column (2), the coefficient of *PledgeR*_*t*_ is 0.0282, which is significantly positive at the 1% level. Comparing the results of Tables 3, 8, we found that the two results are consistent after adopting different measures of financial leverage. This shows that the regression results are robust. The finding supported our H1a that higher stock pledges by ultimate owners promote listed companies to have higher financial leverage.

#### Alternative measure of debt maturity structure

To figure out whether the equity pledge of the ultimate owner affects the debt maturity structure of listed companies, we replaced the debt maturity structure measure with robustness testing. Following Xiao and Liao (2007), we used the ratio of long-term debt to total liabilities as an alternative variable for debt maturity structure. In addition, we employed an incremental variable to express the debt maturity structure variable. This variable is composed of the difference between the value of the debt term structure in year t+1 and the value of the debt term structure in year t, denoted by Δ*DebtS*. Table 9 shows the regression results for Model 2.

**Table 9:** Robustness: the regression results of changing debt maturity structure variables.

	**(1)**	**(2)**
**Variables**	**ΔDebtS_t+1_**	**ΔDebtS_t+1_**
PledgeR_t_	0.0528***	0.0512***
	(6.971)	(6.995)
Size_t_		0.0028**
		(2.266)
ROE_t_		0.0018
		(1.071)
Growth_t_		0.0001
		(1.067)
Cash_t_		−0.0018***
		(−3.379)
NDA_t_		0.0000
		(0.116)
First_t_		−0.0032
		(−0.784)
LRate_t_		−0.0005***
		(−3.629)
Constant	0.0058*	−0.0171
	(1.750)	(−0.865)
Industry	Yes	Yes
Year	Yes	Yes
*N*	18,400	18,400
*R* ^2^	0.231	0.419

Robust t-statistics adjusted for firm level clustering are given in brackets. ***, **, * denote the coefficient is significant at the 1, 5, and 10% level, respectively.

In Table 9, column (1) shows the results of the regression without considering the control variables. In column (1), the coefficient of *PledgeR*_*t*_ is 0.0528, which is significantly positive at the 1% level. Moreover, in column (2), the coefficient of *PledgeR*_*t*_ is 0.0512, which is significantly positive at the 1% level. Comparing the results of Tables 4, 9, we found that the two results are consistent after using different measures of debt maturity structure. This shows that the regression results are robust, and these findings support our hypothesis that increased stock pledges by ultimate owners promote longer debt maturities for listed companies.

## Conclusions and discussions

### Discussions

We examined the impact of share pledges by ultimate owners on corporate debt policy. In emerging capital markets, a high concentration of ownership and separation of cash flow rights and control are common. Equity pledges are prevalent in an environment where private firms face higher financing constraints. We supposed that the tunnel effect and the pressure effect of equity pledges have different effects on corporate debt policy. The results showed that the tunnel effect is supported by the fact that the share pledge ratio of the ultimate owner is positively related to both the financial leverage and debt maturity of the firm, which is consistent with Du and Dai's (2005) findings on the leverage increasing effect of the separation of cash flow rights and control rights.

Further research found that the risk transfer motive of ultimate owners with high personal leverage after pledging equity, rather than the motive to avoid the transfer of control, is an influential mechanism driving the transfer of personal risk to the firm. In addition, previous literature has found that debt structure is an important factor in debt tax protection (Kovacova et al., 2022), while this paper found that debt structure also affects corporate risk-taking capacity. Moreover, previous literature has examined a firm life cycle perspective and found that firms provide distorted financial statements to obtain more favorable debt covenants (Durana et al., 2021). In contrast, this paper extended the study of firms' debt policy choices from the perspective of large shareholders' personal leverage risk. Furthermore, previous literature has found that firms' disclosure of negative news such as environmental penalties increases the cost of debt in the following year (Ding et al., 2022), while this paper considered debt policy from the perspective of corporate agency problems. Additionally, investors can monitor the structure of a firm's financial resources through debt indicators (Valaskova et al., 2021), and this paper provided some references for creditors to capture a firm's financial risk.

### Conclusions

This paper empirically tested the impact of ultimate owner equity pledges on corporate debt policy by using data for Chinese A-share listed companies from 2006 to 2020 to reveal the mechanism of transmission of individual shareholder risk to the firm. The results of this paper found that the pledge of the ultimate controller's equity prompts the expansion of the firm's debt size and longer debt maturity structure, which confirms the tunnel effect hypothesis. Further, this paper uncovered that the high personal leverage of the ultimate controller is an inherent mechanism that induces shareholders to transfer personal risk to the firm, which increases the firm's risk of debt default and validates the risk transfer hypothesis. In detail, even if a firm's actual debt ratio is higher than the target debt ratio, equity pledges induce listed firms to increase their financial leverage and debt maturity, making the firm take excessive debt risk and transfer the risk to creditors. Finally, the results of this paper still hold after endogeneity and robustness tests.

This paper has the following management insights and policy implications. It provides a reference for studying shareholder behavior in emerging markets. The findings suggest that investors should pay close attention to the risk appetite of large shareholders to better understand corporate financial decisions and risk profiles. In addition, in emerging capital markets, private interest in control is high and equity is highly concentrated in the ultimate owners. When the ultimate owners face liquidity shortages, stock pledges become an important tool to alleviate the liquidity shortage. However, this can exacerbate agency problems and harm the interests of minority shareholders and creditors. Finally, these findings enrich the literature on the economic consequences of equity pledges by large shareholders and can serve as an important reference for the design of corporate control mechanisms and financial policies.

This paper has the following limitations and perspectives, such as it only explores individual shareholder risk transfer based on equity pledges, which is a personal financial profile of shareholders. Future research can further explore shareholders' risk preferences in terms of other financial profiles of shareholders, such as portfolio characteristics, to assess the likelihood of their risk transfer. Moreover, considering social and cultural factors, the personal traits of shareholders may influence shareholders' risk preferences, and future research could explore this in the context of multiple characteristics of shareholders. Finally, this paper argues that creditors may be affected by shareholder risk transfer, and future research could further investigate the economic consequences of shareholder risk transfer from the perspective of outside investors.

## Data availability statement

The original contributions presented in the study are included in the article/supplementary material, further inquiries can be directed to the corresponding author/s.

## Author contributions

JX: conceptualization, data curation, formal analysis, investigation, software, and writing—original draft. ZW: conceptualization, funding acquisition, investigation, supervision, and writing—review and editing. RZ: conceptualization, investigation, and supervision. XC: conceptualization, software, validation, methodology, and writing—review and editing. RZ: conceptualization, data curation, and writing—review and editing. All authors contributed to the article and approved the submitted version.

## Funding

This study was financially supported by the National Natural Science Foundation of China (grant numbers: 72211530057, 72173057, and 71672077), National Social Science Fund from National Office for Philosophy and Social Sciences, China (grant number: 20BGL076), Natural Science Foundation of Guangdong Province, China (2021A1515011536), and Fundamental Research Funds for the Central Universities (19JNKY08).

## Conflict of interest

The authors declare that the research was conducted in the absence of any commercial or financial relationships that could be construed as a potential conflict of interest.

## Publisher's note

All claims expressed in this article are solely those of the authors and do not necessarily represent those of their affiliated organizations, or those of the publisher, the editors and the reviewers. Any product that may be evaluated in this article, or claim that may be made by its manufacturer, is not guaranteed or endorsed by the publisher.

## Footnotes

^1^In 2019, China's economy has undergone substantial adjustment, and some well-known private enterprises are facing the dilemma of bankruptcy and reorganization, including HNA Group, etc. High leverage caused by pledging shares and operation difficulties under diversified expansion has pushed the groups into debt crises.
